# KD025 (SLx-2119) suppresses adipogenesis at intermediate stage in human adipose-derived stem cells

**DOI:** 10.1080/21623945.2019.1590929

**Published:** 2019-03-23

**Authors:** Duy Trong Vien Diep, Khue Ha Minh Duong, Hojung Choi, Hee-Sook Jun, Kwang-Hoon Chun

**Affiliations:** Gachon Institute of Pharmaceutical Sciences, College of Pharmacy, Gachon University, Incheon, Republic of Korea

**Keywords:** KD025, SLx-2119, adipogenesis, differentiation, ROCK, Rho-associated kinase

## Abstract

Rho-associated kinases (ROCKs) have been reported to antagonize adipocyte differentiation, and inhibition of ROCKs by small molecules promotes adipogenesis. Surprisingly, our recent study revealed that the ROCK2-specific inhibitor KD025 (SLx-2119), suppresses differentiation at the intermediate stage in 3T3-L1 preadipocytes. To address whether the anti-adipogenic activity of KD025 is a generalizable property, we examined the effect of KD025 in human adipose-derived stem cells (hADSCs). KD025 significantly suppressed the adipocyte differentiation of hADSCs with downregulation of the protein and mRNA expression of various adipogenic and lipogenic markers, including PPARγ, C/EBPα, SREBP-1c, Glut4 and FABP4. Notably, we observed that adipocyte differentiation is effectively suppressed by exposure to KD025 during the mid-to-late period of adipogenesis but not at the earlier stages, showing stage-specificity. Contrary to expectations, KD025 upregulated the insulin signaling, as confirmed by the increased phosphorylation levels of Akt and GSK-3α/β, and the differentiation-promoting activity of insulin signaling was observed to be overwhelmed by the inhibitory activity. In addition, we observed that other ROCK inhibitors (Y-27632, fasudil, and H-1152P) did not suppress but promoted adipocyte differentiation. These results indicate that KD025 suppresses adipocyte differentiation by modulation of key factors activated at the intermediate stage of differentiation, and not by inhibition of ROCK2.

## Introduction

Fats are highly efficient energy sources, and adipose tissue has evolved as the major lipid storing organ in mammals. Interestingly, beside energy storage, diverse metabolic activities of adipose tissue have been reported in recent years, as an endocrine organ responsible for the synthesis and secretion of various hormones and cytokines ^[1].^ To maintain the energy balance, the metabolism in adipose tissue should be regulated tightly, and mammals have developed intricate mechanisms to store and utilize fats. The balanced metabolic control of fat tissues is critical in leading a healthy lifestyle as can be seen from the fact that its dysfunction is a leading cause of obesity, type 2 diabetes, and various other metabolic diseases [2].

Adipogenesis is a complex process, progressing from precursor stem cells to adipocytes, which can be divided into 2 phases: determination and terminal differentiation [3,4]. The terminal differentiation process is relatively well understood compared to the determination phase. During adipogenesis, various gene expression events are involved in the regulation of morphology and physiology [4]. Peroxisome proliferator-activated receptor γ (PPARγ) and CCAAT enhancer-binding protein α (C/EBPα) are essential regulators of adipogenesis, with PPARγ being the proximal effector of this process [5]. Upon activation, these two factors induce the expression of metabolic genes associated with adipocyte phenotype, such as glucose transporter 4 (GLUT4), fatty acid binding protein 4 (FABP4), and leptin [6]. Throughout these events, lipid droplets start to appear in the cytoplasm and cells take on the characteristics of mature adipocytes [7].

Rho-associated coiled-coil-containing protein kinases (ROCKs/Rho-kinases) are serine/threonine kinases introduced as downstream effectors of the small GTPase Rho [8–10]. There are two isoforms of ROCK: ROCK1 (ROKβ) and ROCK2 (ROKα); their structures are conserved with 65% of overall amino acid identity and 92% in the kinase domain [10]. ROCKs are key players in various cellular events such as actin cytoskeleton organization, cytokinesis, differentiation, apoptosis, glucose metabolism, cell adhesion/motility, and inflammation. Hence, attention has focused on the development of ROCK-targeting agents for decades, although only fasudil (HA-1077) and ripasudil are approved drugs as therapeutics in Japan and China [9]. A set of evidences strongly indicate that the Rho/ROCK signaling pathway regulates adipocyte differentiation negatively, as suggested by: i) rounded cell morphology and loss of actinomyosin fiber formation, a process in which Rho-ROCK inactivation is a prerequisite [11–13], ii) the modulation of Rho-ROCK activity by ectopic expression of constitutively active Rho, p190B RhoGAP-deletion, and pan-inhibitors (Y-27632 and fasudil) showing anti-adipogenic activity of Rho-ROCK pathway [11,13,14], and iii) especially, ROCK2, but not ROCK1, is suggested as the responsible isoform that delivers the Rho’s anti-adipogenic activity in 3T3-L1 and mouse embryonic fibroblasts (MEFs) [14].

KD025 (SLx-2119) is a ROCK2-specific inhibitor having an IC_50_ = 105 nM for ROCK2 (IC_50_ = 24 µM for ROCK1) [15]. KD025 is currently under clinical development as a treatment for diverse diseases. It has been shown to dilate carotid arteries and parenchymal arterioles in mice [16]. Other studies have shown that oral administration of KD025 to human subjects ameliorates immune homeostasis by downregulating the autoimmune response in human T cells [17,18]. In a previous study, we demonstrated that KD025, but not other ROCK inhibitors, suppresses adipocyte differentiation of the 3T3-L1 pre-adipocyte cells by down-regulating the expression of key adipogenic/lipogenic genes such as PPARγ, C/EBPα, FABP4 and Glut4 [19]. Furthermore, we also demonstrated that KD025 regulates a certain key pro-adipogenic factor which is active during the intermediate stage, and that the mechanism is irrelevant to the Rho-ROCK pathway.

Despite these findings, more evidences are required to clearly ascertain the effect of KD025, since the effect of KD025 on adipocyte differentiation is contrary to other ROCK inhibitors. This study was therefore undertaken to investigate whether the anti-adipogenic effect of KD025 is reproducible in stem cell differentiation. We introduced the adipocyte differentiation system using human adipose-derived stem cells (hADSCs) as an experimental model for following reasons: first, as hADSCs are derived from humans, we would be able to establish if the effect of KD025 is species-specific or general; second, they are not fate-determined yet, but have the potential to differentiate into multiple cell types according to stimuli, such as adipocytes, osteoblasts, or chondrocytes. Thus, this would enable us to examine the effect on the whole process related to adipogenesis (fate determination and terminal differentiation), which is similar to the *in vivo* environment. This study shows that KD025 suppresses the adipogenesis of human stem cells, thereby confirming evidences that KD025 interrupts the intermediate stage during adipogenesis.

## Materials and method

### Culture of hADSCs

**Figure 1.**
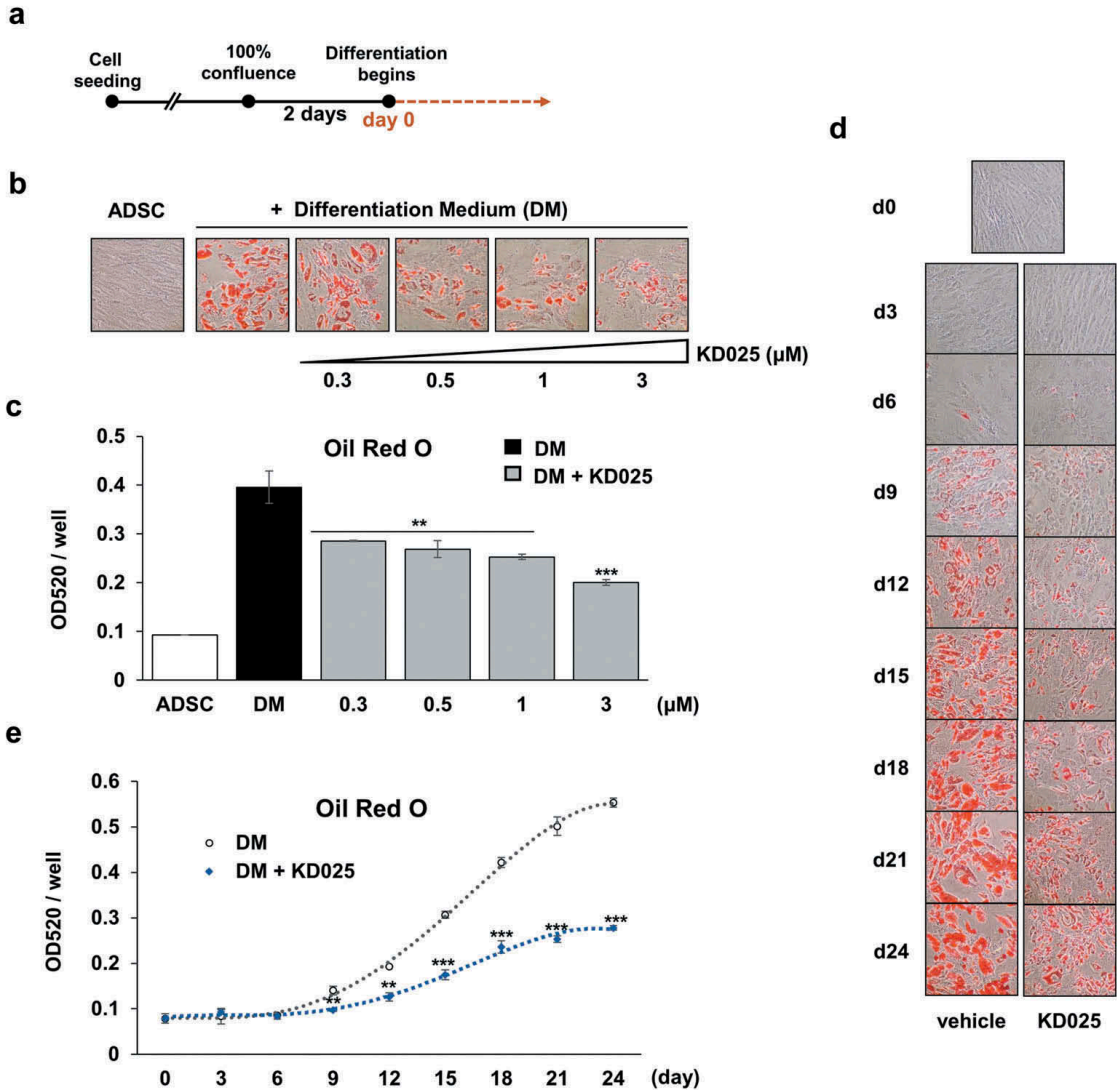
Measurement of the effect of KD025 on adipogenesis in hADSCs. hADSCs were differentiated by culturing in differentiation media (DM) with or without KD025 at the indicated concentrations. (a) Experimental scheme of differentiation. (b) Cells were stained with ORO at day 15, and microscopic images were taken after the start of differentiation (indicated as day 0). Cells were exposed to 0.3, 0.5, 1, and 3 μM of KD025. (c) Lipid accumulation of (b) was assessed by measuring absorbance at 520 nm. (d) Cells were differentiated with or without 3 μM KD025 until day 24. Microscopic pictures of cells are presented. (e) Lipid accumulation of (d) was assessed by measuring absorbance at 520 nm. **p < 0.01; ***p < 0.001 *vs*. control.

hADSCs (cat#R7788-115, Invitrogen, CA, USA) were cultured in MesenPro RS™ Medium (Invitrogen, CA, USA) supplemented with 100 units/mL of penicillin and 100 μg/mL of streptomycin (Cellgro, VA, USA), in a humidified incubator at 37°C and 5% CO_2_. In order to differentiate into adipocytes, cells were grown for 2 days of post-confluence, after which the medium was replaced with StemPro® Adipogenesis Differentiation Medium (Life technologies, CA, USA). Media was changed every 3 days during the specific periods of cultivation. The differentiation scheme is depicted in Figure 1(a).

### ROCK inhibitors

KD025 and fasudil were purchased from MedChem Express (NJ, USA), Y-27632 from Selleck Chemicals (TX, USA), and H-1152P from R&D Systems (MN, USA).

### RNA isolation

Total RNA was extracted by Trizol (Invitrogen), according to the protocol suggested by the manufacturer. Briefly, cells were lysed with an appropriate amount of Trizol, followed by addition of chloroform and 5-minute incubation at room temperature. The sample was then centrifuged for 15 min at 12,000 x *g* at 4°C, resulting in separation of a lower red phenol-chloroform layer, an interphase, and an upper aqueous phase. The aqueous phase was gently separated and transferred to a new tube, to which isopropanol was added, and the mixture was incubated for 10 min. The sample was then centrifuged for 10 min at 12,000 x *g* at 4°C. After discarding the supernatant, the gel-like pellet at the bottom of the tube was washed with 75% ethanol, and centrifuged at 7500 x *g* for 5 min. The final pellet was collected, air-dried, and then diluted in an appropriate amount of RNase-free water. The concentration of RNA was measured by the NanoDrop™ 2000c spectrophotometer (Thermo Fisher Scientific, MA, USA).

### Quantitative RT-PCR

For reverse transcription, cDNA was acquired from 500 ng of total RNA using the SuperScript First-strand Synthesis System (Invitrogen). The acquired cDNA was analyzed by quantitative RT-PCR using SYBR Green TOPreal qPCR 2X PreMix (Enzynomics, Daejeon, Korea) with an applied Biosystems Mx3005P qPCR instrument (Applied Biosystems, CA, USA). The relative expressions of genes of interest were calculated using the 2^−ΔΔCt^ method. Sequences of primers used for qRT-PCR are listed in Table 1.

**Table 1.** List of real time PCR primers and sequences.

Type		Gene	Primer	Primer Sequence (5ʹ-3ʹ)
Adipogenic genes	**Early gene**	***CEBPB***	forward	CGCTTACCTCGGCTACCA
			reverse	ACGAGGAGGACGTGGAGAG
	**Key genes**	***CEBPA***	forward	GGAGCTGAGATCCCGACA
			reverse	TTCTAAGGACAGGCGTGGAG
		***PPARG***	forward	TCCATGCTGTTATGGGTGAA
			reverse	TGTGTCAACCATGGTCATTTC
Lipogenic genes		***SREBF1***	forward	CACAACGCCATTGAGAAGC
			reverse	GCGCAAGACAGCAGATTTAT
		***SLC2A4***	forward	GGCATGGGTTTCCAGTATGT
			reverse	GCCTCGAGTTTCAGGTACTC

### Western blot analysis

Cells were suspended in lysis buffer (20 mM tris pH 7.5, 5 mM EDTA, 10 mM Na_4_P_2_O_7_, 100 mM NaF, 2 mM Na_3_VO_4_, 1% NP-40, 1 mM PMSF, 10 μg/mL aprotinin, and 10 μg/mL leupeptin) and 20 μg lysate was separated by SDS-PAGE. The fractionated proteins were then transferred from gel to nitrocellulose membrane. The membranes were incubated with primary antibodies [FABP4, PPARγ, and Akt were from Santa Cruz Biotechnology (TX, USA); phospho-Ser^21/9^ GSK-3α/β, phospho-Ser^4,7,3^,kt and phospho-Thr^308^^^[8]^^Akt were from Cell Signaling Technology (MA, USA), and β-tubulin from Abcam (MA, USA)], after which the probed membranes were incubated with the secondary antibodies. The resultant bands were detected using SuperSignal West Femto Maximum Sensitivity Substrate (Thermo Fisher Scientific, MA, USA), visualized under the ChemiDoc imaging system (Bio-Rad, CA, USA), and quantified by the Image Lab software (Bio-Rad) and ImageJ software (National Institutes of Health, ver.1.51k, https://imagej.nih.gov/ij/).

### Oil Red O staining

Adipogenic differentiation was visualized using Oil Red O (ORO) (Sigma-Aldrich, MA, USA). Differentiated cells were washed with PBS and incubated with 10% formalin for 5 min. Cells were then fixed with the same volume of fresh formalin for at least 1 hr. After fixing, cells were rinsed with 60% isopropanol, air-dried completely, and stained with ORO working solution (6 parts of 1% ORO stock in 100% isopropanol mixed with 4 parts of distilled water; let sit at room temperature for 20 min and filter through 0.2 μm) for 10 min. Stained cells were washed with water 4 times. Pictures of stained cell were taken by a microscope (Nikon Eclipse TS100-F, Japan). In order to quantify the intensity of ORO, the dye was extracted by adding 100% isopropanol and incubated for 10 min. Absorbance was measured by the Synergy H1 hybrid reader (BioTek, VT, USA) at 520 nm.

### Measurement of total cell number

For counting total number of cells, hADSCs were seeded in 12-well plates (~2 x 10^5^ cells/well). The cells were cultured until reaching 100% confluence, and further incubated for 2 days after which they were differentiated with or without 3 μM KD025. Following differentiation, cells were trypsinized, and the total number of cells was counted at indicated time points (days 0, 3, 9, 15) using a hemocytometer.

### Statistical analysis

Data are expressed as means ± SEM or SD. Student’s t-test was used to compare between means. All experiments were performed in triplicate. Differences are accepted as significant at p < 0.05.

## Results

### KD025 inhibits adipocyte differentiation in hADSCs

In our previous study, we demonstrated that KD025 attenuates adipogenesis in 3T3-L1 cells committed to preadipocytes [19]. This study further examined the effect of KD025 on adipogenesis in uncommitted, multipotent hADSCs. Preliminary tests revealed that differentiation was clearly recognized at day 15 after stimulation, both by microscopic observation and colorimetric evaluation using Oil Red O (ORO). To examine the effect of KD025 on adipogenesis, cells were seeded and grown to 100% confluency and maintained for 2 days additionally. The cells were treated with or without KD025 at varying concentrations (0.3, 0.5, 1 and 3 μM) during adipogenesis, and cells were stained with ORO on day 15. Microscopic observation revealed that KD025 dose-dependently inhibited the lipid accumulation in hADSCs (Figure 1(b)). Consequently, the level of ORO stained, measured spectrophotometrically, was lower in the KD025-treated groups as compared to vehicle-treated control (Figure 1(c)). Especially, treatment with 3 μM KD025 decreased the lipid accumulation by ~50% compared to the control, without any visible toxicity. Hence, all subsequent experiments were performed at this concentration.

The effect of KD025 on the differentiation process was observed every three days, from day 3 to day 24, after differentiation stimulation. In the vehicle treated cells, the ORO-staining revealed significant accumulation of fat on day 9 with gradual increase during differentiation, with subsequent decrease in this increment till day 24 (Figure 1(d,e)). In contrast, KD025 exposure significantly inhibited the lipid accumulation, and this effect was much more prominent at later time points (from day 18 onwards).

**Figure 2.**
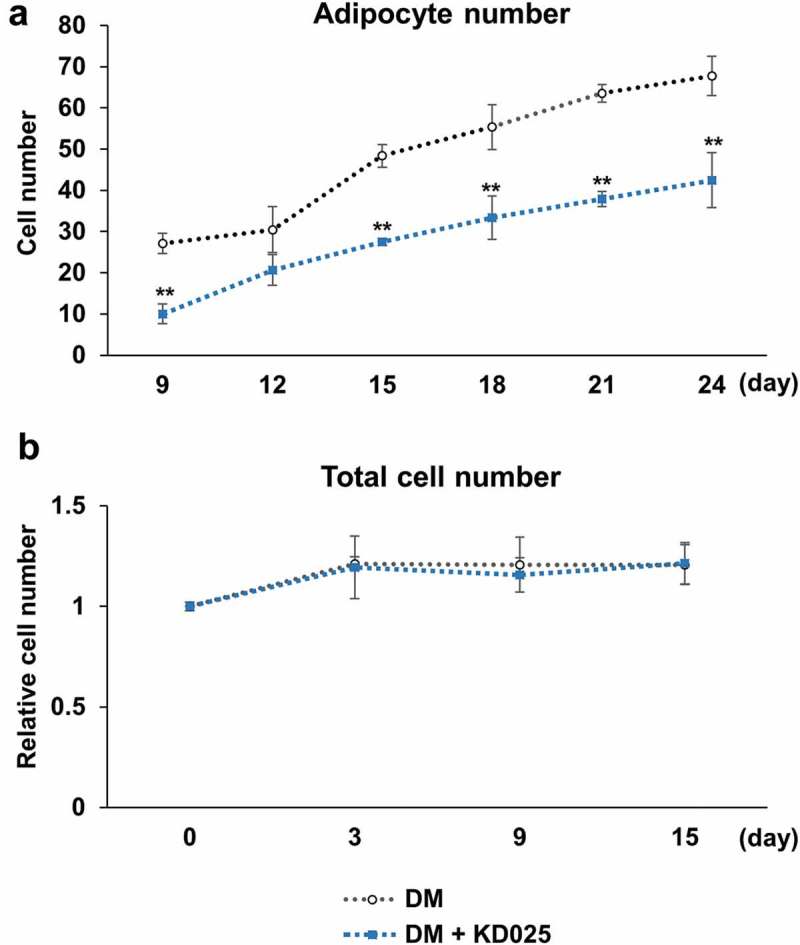
Effect of KD025 on fate determination of human stem cells. hADSCs were differentiated with or without 3 μM KD025 for the indicated periods. (a) The number of ORO-stained cells, which are considered adipocytes, were counted in each group from 30 different areas of the obtained microscopic images. (b) All cells were trypsinized, and total number of cells was counted using a hemocytometer. The total number of cells at each time point was expressed as a ratio to the total cell number at the differentiation starting point. *p < 0.05, **p < 0.01; ***p < 0.001 vs. control.

A decrease in fat mass indicates inhibition in the maturation of adipocytes by KD025 treatment. Data in Figure 1(a,c) shows that the amount of fat per cell is lesser in KD025-treated cells, indicating that the biosynthetic activity of fat in each cell is low. However, these results do not provide information whether there is any reduction in the number of differentiated cells. Therefore, we further examined the effect of KD025 on the fate-determining ability of the cell by counting the differentiated cells in each group. At each indicated time point, ORO-stained adipocytes were counted in multiple areas of the microscopic images. Similar to the effect of lowering fat biosynthesis, exposure to KD025 significantly decreased the number of cells differentiating into adipocytes during the course of differentiation, by ~30% on day 24 (Figure 2(a)). To evaluate if this change in adipocyte number is due to a decrease in total cell number, we trypsinized and counted all cells using a hemocytometer. As presented in Figure 2(b), the number of cells increased by ~20% by day 3 and was similarly maintained in both groups thereafter, indicating that cell proliferation continued during the differentiation process, although the magnitude was not as much when compared to 3T3-L1 cells described in the previous study [19]. Moreover, treatment with 3 μM KD025 had no effect on the total cell number during adipogenesis. These results indicate that KD025 prevents both the differentiation event and subsequent fat biosynthesis without affecting cell proliferation which is the early differentiation event.

### Treatment of KD025 down-regulates the expression of key adipogenic and lipogenic genes

**Figure 3.**
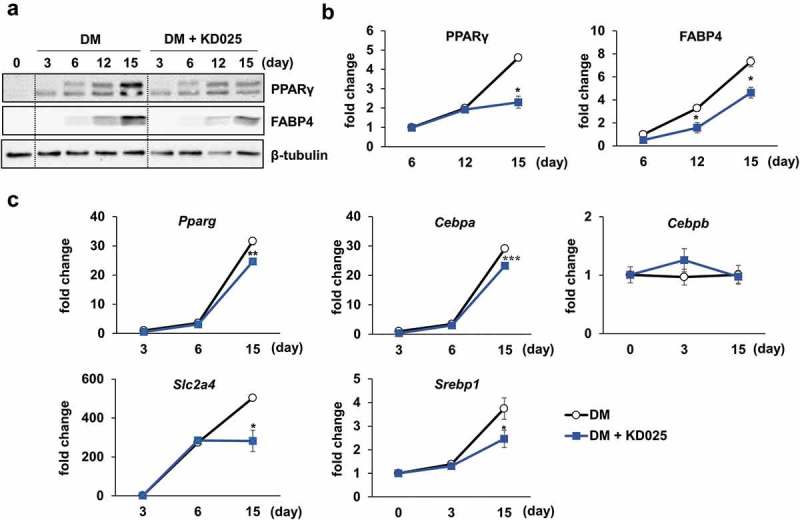
Effect of KD025 on adipogenic and lipogenic markers. hADSCs were differentiated by incubating in DM with or without 3 μM KD025 over 15-day period. (a) The protein expression levels of PPARγ and FABP4 were analyzed by Western blot, at the indicated time points. β-tubulin was used as a loading control. (b) The expression levels of PPARγ and FABP4 was quantified using the ImageJ software. The relative level was assessed by fold changes compared to day 6/KD025-untreated control cells. (c) The mRNA expression levels of adipogenic genes (*PPARG, CEBPA, CEBPB*) and lipogenic genes (*SLC2A4, SREBF1*) at the indicated time points. The relative level was assessed by fold changes compared to KD025-untreated control cells at day 0. *p < 0.05, **p < 0.01; ***p < 0.001 vs. corresponding control condition.

To evaluate the effect of KD025 on adipogenesis, hADSCs were differentiated in the presence of KD025, and subsequently collected on days 3, 6, 12 and 15. Western blot analysis revealed that the protein expression levels of PPARγ and FABP4 increased gradually during differentiation until day 12 and were highly expressed on day 15, indicating day 15 to be a late stage. The PPARγ protein levels were not changed by KD025 with any significance in the early-to-mid stage (days 6 and 12), but suppressed significantly on day 15 (Figure 3(a,b)). In contrast, the suppressive effect on the expression level of FABP4 was significant from the early stage and sustained to the late stage. qRT-PCR was applied to further examine the transcriptional levels of key adipogenic (*PPARG, CEBPA*, and *CEBPB*) and lipogenic markers (*SREBF1* [Sterol regulatory element binding transcription factor 1, SREBP1] and *SLC2A4* [Solute carrier family 2 member 4, Glut4]) during the course of adipogenic differentiation. Compared to the vehicle-treated group, KD025 significantly suppressed the expression of *PPARG, CEBPA, SREBF1, SLC2A4* on day 15 (Figure 3(c)). However, no significant change was detected in the expression levels of the early adipogenic gene *CEBPB*. These results indicate that KD025 possibly modulates a specific stage of differentiation.

### Stage-specific effect of KD025 on adipogenesis of hADSC

**Figure 4.**
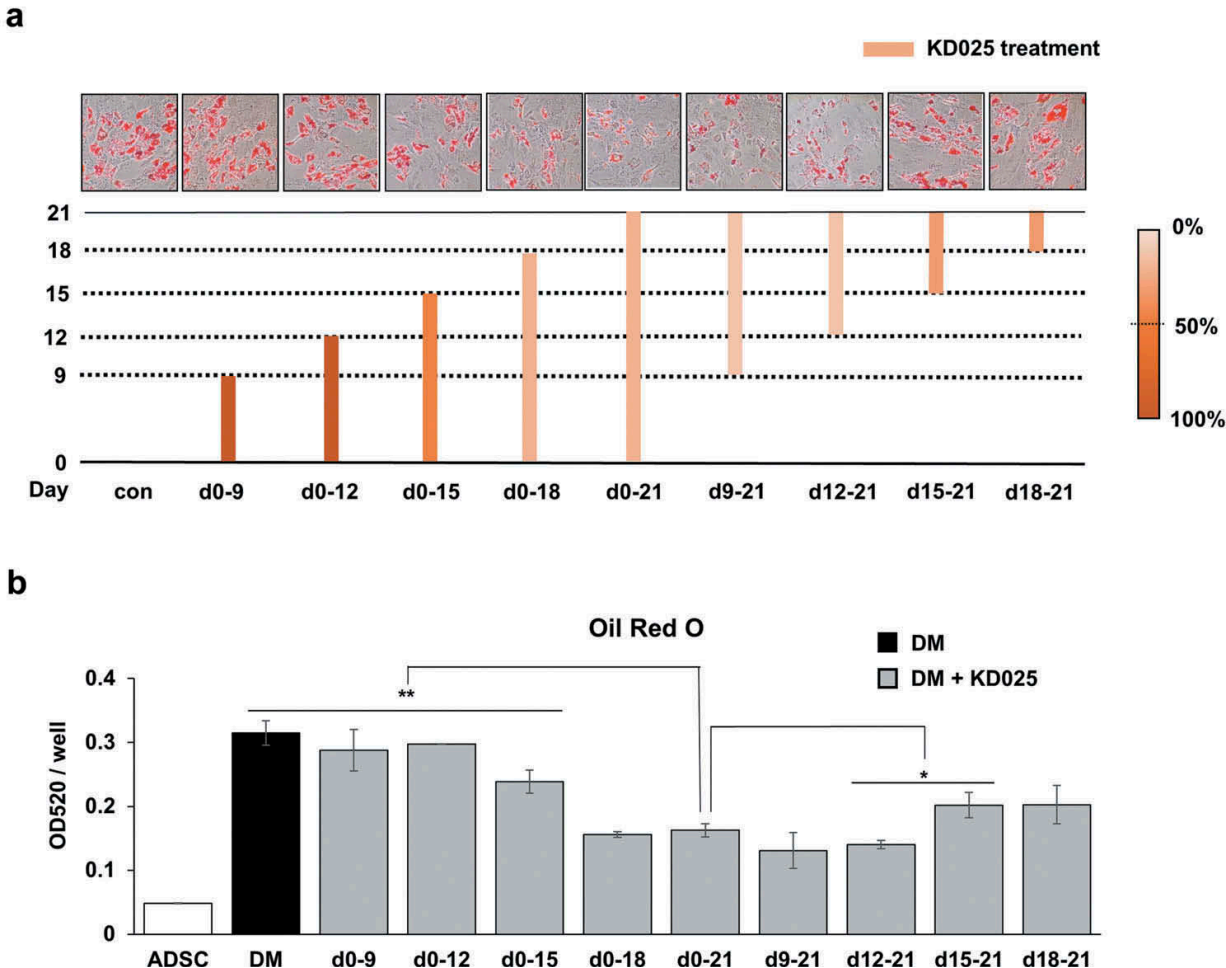
Stage-specific effect of KD025 on adipogenesis of hADSC. (a) During differentiation of hADSCs, cells were exposed to 3 μM KD025 at various time schedules as indicated, and stained with ORO. The time schedules of inhibition are denoted as the corresponding rods, and the amount of lipid measured was indicated by the relative darkness of the color compared to DM treated group as 100%.(b) Lipid accumulation was assessed by measuring absorbance at 520 nm. The positive control treated for the entire period (from day 0 to day 21) is marked in orange. *p < 0.05, **p < 0.01 vs. positive control (orange box).

The absence of inhibitory effects in early events (cell proliferation and adipogenic gene expression at early stage) indicates that the molecular target of KD025 may be attaining functionality at the mid-to-late stage. To examine this hypothesis, we examined the stage at which KD025 becomes functional during the 21 days of cell differentiation. The group treated with KD025 during the entire 21-day period (day 0–21) had ~50% lower lipid contents (Figure 4(a,b)). Compared to this group, no significant differences were observed when the drug was not treated only at the beginning (treated from day 9 to 21 or from day 12 to 21) or end (treated from day 0 to 18) of the whole period. These results indicate that KD025 exerts its effects on the molecular target between days 12 and 18. Besides this, no effect was observed after treatment with KD025 during the early period (days 0–9 and days 0–12), while a relatively weak change was detected with treatment from days 0–15. These results further imply that the molecular target of KD025 might be functionally inactive until day 12, but gradually attains active functionality thereafter. Besides, between days 15–21 and between days 18–21, KD025 was observed to partially suppress adipogenesis. Taken together, our results indicate that the target of KD025 becomes activated in the mid-to-late stage of adipogenesis, especially from day 12 to 18.

### Effect of KD025 on the insulin signaling pathway

**Figure 5.**
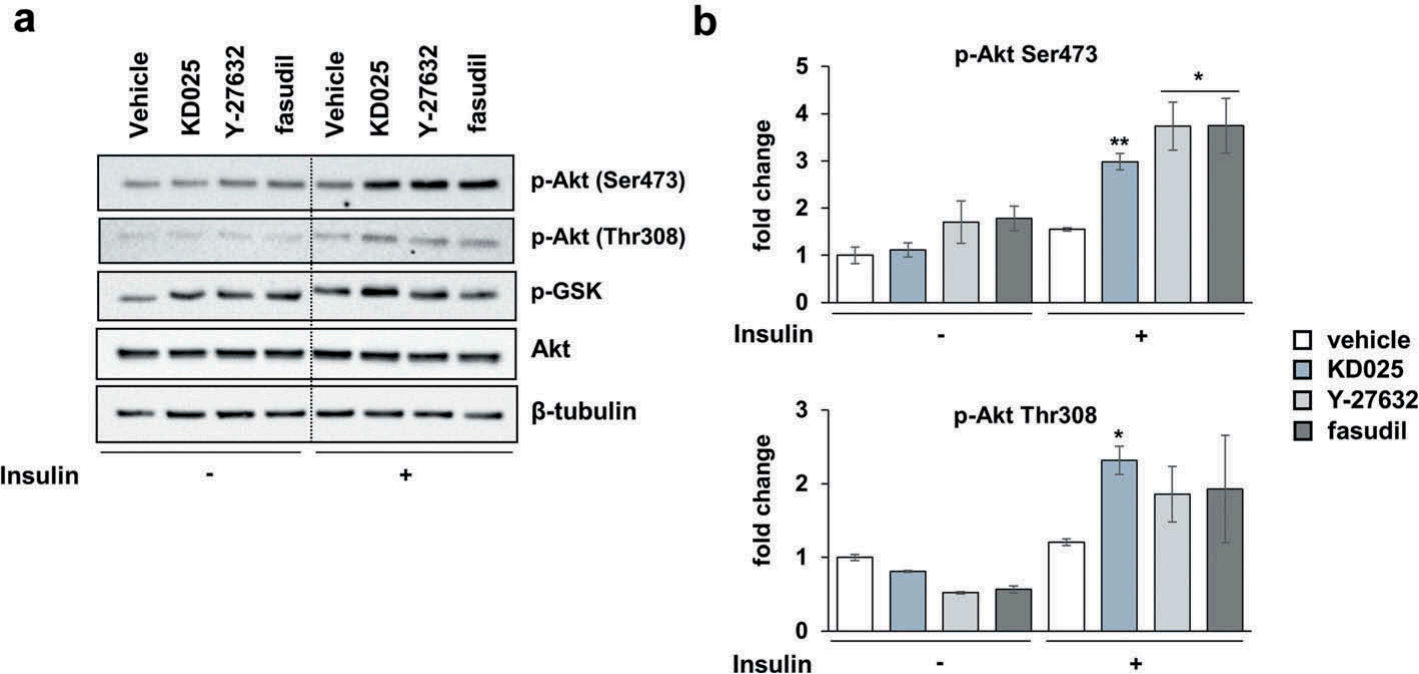
Effects of ROCK inhibitors on insulin signaling. hADSCs were starved in serum-free medium and then stimulated with 20 nM insulin for 20 min. ROCK inhibitors were treated during starvation and stimulation periods. Western blot was performed to evaluate the level of p-Akt (Ser473), p-Akt (Thr308), Akt, and p-GSK-3α/β. β-tubulin was used as the loading control. (b) Band intensity of p-Akt (Ser473) and p-Akt (Thr308) was quantified using the Image J software. The relative level was assessed as fold changes compared to the unstimulated, vehicle-treated control cells. *p < 0.05, **p < 0.01 vs. control.

Since insulin plays an important role in adipogenesis, we investigated whether KD025 regulates adipogenesis by interfering the insulin signaling pathway. To see whether the effect of KD025 was mediated by the inhibition of insulin action in hADSCs, we examined the insulin signaling pathway by stimulating starved cells with insulin (20 nM) in the presence or absence of KD025. Western blot analysis showed that the exposure to KD025 augmented the phosphorylation of Akt both at Ser473 and Thr308 residues; this effect was especially distinct under insulin stimulation (Figure 5(a,b)). KD025 also up-regulated the phosphorylation of GSK-3α/β, a known substrate of Akt. The ROCK inhibitors Y-27632 and fasudil, also enhanced Akt-mediated insulin signaling. These results indicate that cell differentiation is inhibited despite the enhancement of Akt-mediated insulin signaling, suggesting that the target of KD025 may be a critical regulator, the activation of which is a prerequisite for the pro-adipogenic action of the insulin-Akt pathway.

### Anti-adipogenic activity of KD025 might not be mediated by ROCK inhibition

**Figure 6.**
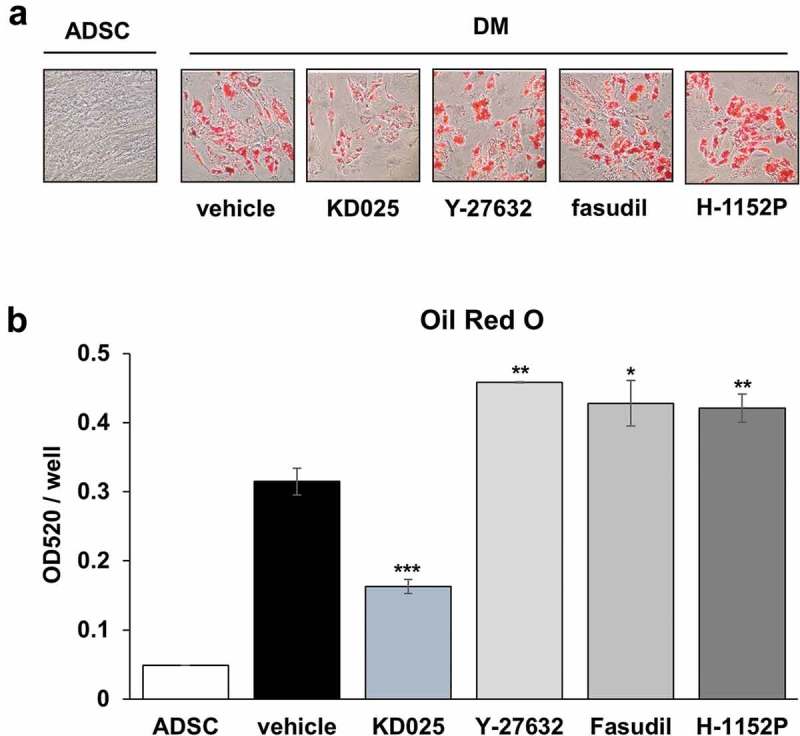
Effects of ROCK inhibitors on adipogenesis of hADSCs. (a) During differentiation, cells were exposed to KD025 (3 µM), Y-27632 (5 µM), fasudil (5 µM) and H-1152P (3 µM). Cells were then stained with ORO on day 15. (b) Lipid accumulation was assessed by measuring absorbance at 520 nm. *p < 0.05, **p < 0.01; ***p < 0.001 vs. untreated.

**Figure 7.**
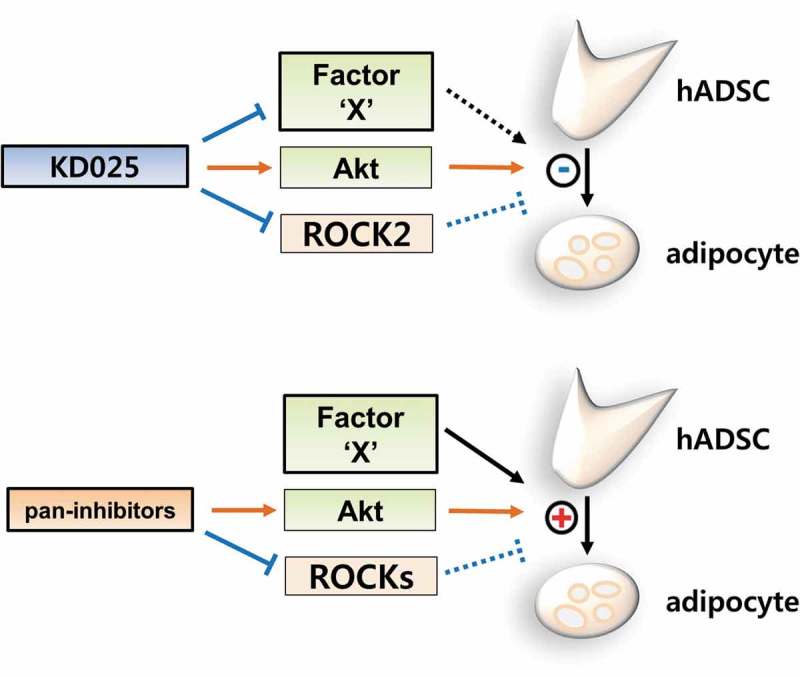
Proposed model of the mechanistic action of KD025 on the differentiation of hADSCs. KD025 inhibits adipocyte differentiation in hADSCs by suppressing a certain pro-adipogenic regulator. This inactivation overwhelms the pro-adipogenic activity resulting from the Akt activation and ROCK inhibition. Pan-inhibitors promote adipogenesis by activating Akt and suppressing ROCKs.

Previously, pan-inhibitors (such as Y-27632 and fasudil) were shown to enhance adipocyte differentiation [13,14,22]. By applying the 3T3-L1 model, our studies distinguished KD025 from other ROCK inhibitors with regards to the anti-adipogenic effect [19]. To confirm that the anti-adipogenic effect of KD025 is not species-specific but general, we examined the effect of several ROCK inhibitors on adipogenesis using hADSCs. Cells were differentiated in the presence of KD025, Y-27632, fasudil, and H-1152P. H-1152P is a potent ROCK2 inhibitor (K_i_ (ROCK2) = 1.6 nM) [20]. As shown in Figure 6(a,b), unlike KD025, lipid accumulation in hADSCs was not inhibited after exposure to all three ROCK inhibitors. In contrast, these inhibitors enhanced the adipogenesis of hADSCs, which is in good agreement with the results showing increase in insulin signaling (Figure 5). Collectively, these findings suggest that the inhibitory effect of KD025 on adipogenesis might be mediated by suppression of a key adipogenic factor which is independently regulated by ROCK. The proposed mechanistic model of KD025 is presented in Figure 7.

## Discussion

For decades, ROCK inhibitors have been investigated in clinical trials for a wide range of diseases such as glaucoma, psoriasis vulgaris, fibrosis, and erectile dysfunction [9,23]. Recently, the isoform-specific metabolic functions of ROCKs have been receiving greater attention [24–27]. Numerous evidences also suggest ROCKs to be negative regulators in adipocyte differentiation. Noguchi M. *et al*. described that ROCK2, but not ROCK1, is the responsible anti-adipogenic ROCK in 3T3-L1 and MEF models [14]. In particular, Y-27632 and fasudil have been shown to promote adipocyte differentiation [13,14,22]. In contrast to this research, there exists a lack of effort to develop ROCK inhibitors as agents to treat metabolic disorders. Intriguingly, in our previous study, we discovered the anti-adipogenic role of KD025 in the 3T3-L1 mouse cell line. Whereas other ROCK inhibitors promote adipocyte differentiation by inhibiting ROCKs, KD025 was thought to inhibit a certain key regulator of differentiation which can overwhelm the adipogenic effect evoked by inhibition of ROCKs [19]. Notably, this inhibitory effect was not attributed to the inhibition of ROCK2 (the actual target of KD025), but rather to the modulation of a target that regulates the intermediate stage of adipogenesis.

In the current study, we investigated whether the anti-adipogenic activity of KD025 can be perceived as a general characteristic, by evaluating its effect on the differentiation of human stem cells. Our results demonstrate that KD025 consistently inhibits adipocyte differentiation in human stem cells as well as in mouse preadipocytes [19]. It is noteworthy that the effect of KD025 was distinct from other ROCK inhibitors (Y-27632, fasudil, H-1152P) which do not suppress adipocyte differentiation, but rather stimulate according to the context. Along with the reported evidences that ROCKs antagonize adipocyte differentiation, these results assert that a potent adipogenic regulator may be the target of KD025, and that the regulator may be an off-target in terms of other ROCK inhibitors.

Most kinase inhibitors, including KD025, compete with ATP for binding at the enzyme’s active site. The sequences are well conserved among kinases, resulting in a relatively high off-target effect, which leads to polypharmacology [28]. This interpretation therefore implies that the identification of the valid target of KD025 relevant to adipogenesis is essential for the deeper understanding of the precise mechanistic action of KD025.

The 3T3-L1 cells are considered preadipocytes already committed to the adipogenic pathway that undergo the terminal differentiation process [29]. In contrast, the human ADSCs model is not fate-determined, but multipotent mesenchymal stem cells that are able to differentiate into multiple lineages such as adipocytes, chondrocytes, osteoblasts and muscle cells [21,30,31]. From this perspective, hADSCs are a more convincing system in interpreting the physiological effects of KD025 in humans. Despite differences between the two cell types, the observed properties of KD025 are very similar in many aspects: i) In both cases, KD025 interrupts adipogenesis at the intermediate stage, accompanied with efficient suppression of key adipogenic transcription factors such as PPARγ and C/EBPα. Notably, the early stage was not affected, suggesting that KD025 has a specific target which is active for a specific period of time, ii) Other ROCK inhibitors did not suppress adipogenesis but promoted adipogenesis of hADSCs and 3T3-L1 preadipocytes, and iii) KD025 does not affect cell proliferation during adipogenesis at the tested concentrations.

Conversely, there are some inherent differences observed: i) In hADSCs, differentiation is suppressed by KD025 even under potentiation of insulin signaling. In the previous study, we did not observe any significant changes in the insulin action after exposure of 3T3-L1 cells to KD025. These observations imply that KD025 may influence a regulator with dominant functions over insulin signaling, and ii) H-1152P promoted adipogenesis of hADSCs but had no effect on 3T3-L1 preadipocytes. At present, the reason for these differences are unclear. However, all three inhibitors evaluated (Y-27632, fasudil, and H-1152P) were shown to promote adipocyte differentiation in hADSCs, thus supporting the generally accepted theory that ROCKs have anti-adipogenic activity. Above all, considering all ROCK inhibitors, it should be noted that the anti-adipogenic activity may be accepted as a discernible property of KD025.

Along with mouse preadipocytes [19] and hADSCs, KD025 suppresses the mRNA and protein expression of essential adipogenic/lipogenic markers such as PPARγ, C/EBPα, Glut4, SREBP1, and FABP4. C/EBPα and PPARγ are known as the master regulators of adipogenesis that determine the cell fate. Upon activation, PPARγ and C/EBPα act synergistically to activate adipocyte-specific genes, including stearoyl-CoA desaturase-1 (SCD-1), phosphoenolpyruvate carboxykinase (PEPCK), FABP4, and GLUT4 [32]. The significantly decreased expression of these markers after KD025 exposure appears evident in the mid-to-late stage of the adipogenesis process. Besides, treatment with KD025 during the early stage of differentiation only (day 0–12) did not show any effect (Figure 4). Furthermore, the expression of *CEBPB*, an early adipogenic gene, was not altered by KD025 significantly during adipogenesis. All these evidences strongly support that KD025 regulates its target at the intermediate stage of adipogenesis.

The insulin signaling pathway is a potent activator of adipogenesis [4,33,3^4^]. We found that KD025 as well as other inhibitors, increase the phosphorylation of Akt, an essential downstream factor of insulin signaling. The activation of insulin signaling, probably mediated by ROCK inhibition, was unable to override the anti-adipogenic effect mediated by modulation of an unknown regulator. Thus, the anti-adipogenic activity of KD025 might not be mediated through modulation of insulin signaling.

In conclusion, this study demonstrates that KD025 inhibits adipocyte differentiation of hADSCs by suppressing the expression of pro-adipogenic factors. The anti-adipogenic activity of KD025 is driven by targeting unknown factor(s) that overwhelm the pro-adipogenic effect of Akt activation and ROCK inhibition. We speculate that the expected target may be activated during the mid-to-late phase of adipogenesis. Further investigations are required to clarify the unknown target of KD025 which would help explain the underlying mechanism.

## Additional Information

Competing financial interests: The authors declare no competing financial interests.

## References

[1] Coelho M, Oliveira T, Fernandes R. Biochemistry of adipose tissue: an endocrine organ. Arch Med Sci. 2013;9:191–200. 23671428 10.5114/aoms.2013.33181 PMC3648822

[2] Galic S, Oakhill JS, Steinberg GR Adipose tissue as an endocrine organ. Mol Cell Endocrinol. 2010;316:129–139. 19723556 10.1016/j.mce.2009.08.018

[3] Lowe CE, O’Rahilly S, Rochford JJ Adipogenesis at a glance. J Cell Sci. 2011;124:2681–2686. 21807935 10.1242/jcs.079699

[4] Rosen ED, MacDougald OA Adipocyte differentiation from the inside out. Nat Rev Mol Cell Biol. 2006;7:885–896. 17139329 10.1038/nrm2066

[5] Rosen ED, Hsu C-H, Wang X, et al C/EBPα induces adipogenesis through PPARγ: a unified pathway. Genes Dev. 2002;16:22–26. 11782441 10.1101/gad.948702 PMC155311

[6] Cristancho AG, Lazar MA Forming functional fat: a growing understanding of adipocyte differentiation. Nat Rev Mol Cell Biol. 2011;12:722–734. 21952300 10.1038/nrm3198 PMC7171550

[7] Rosen ED, Walkey CJ, Puigserver P, et al Transcriptional regulation of adipogenesis. Genes Dev. 2000;14:1293–1307. 10837022

[8] Amano M, Nakayama M, Kaibuchi K Rho-kinase/ROCK: a key regulator of the cytoskeleton and cell polarity. Cytoskeleton (Hoboken, NJ) 2010; 67:545–554. 10.1002/cm.20472 PMC3038199 20803696

[9] Feng Y, LoGrasso PV, Defert O, et al (ROCK) inhibitors and their therapeutic potential. J Med Chem. 2016;59:2269–2300. 26486225 10.1021/acs.jmedchem.5b00683

[10] Riento K, Ridley AJ ROCKs: multifunctional kinases in cell behaviour. Nat Rev Mol Cell Biol. 2003;4:446–456. 12778124 10.1038/nrm1128

[11] Sordella R, Jiang W, Chen G-C, et al Modulation of Rho GTPase signaling regulates a switch between adipogenesis and myogenesis. Cell. 2003;113:147–158. 12705864 10.1016/s0092-8674(03)00271-x

[12] Jaffe AB, Hall A RHO GTPASES: biochemistry and biology. Annu Rev Cell Dev Biol. 2005;21:247–269. 16212495 10.1146/annurev.cellbio.21.020604.150721

[13] McBeath R, Pirone DM, Nelson CM, et al Cell shape, cytoskeletal tension, and RhoA regulate stem cell lineage commitment. Dev Cell. 2004;6:483–495. 15068789 10.1016/s1534-5807(04)00075-9

[14] Noguchi M, Hosoda K, Fujikura J, et al Genetic and pharmacological inhibition of Rho-associated kinase II enhances adipogenesis. J Biol Chem. 2007;282:29574–29583. 17681946 10.1074/jbc.M705972200

[15] Boerma M, Fu Q, Wang J, et al Comparative gene expression profiling in three primary human cell lines after treatment with a novel inhibitor of Rho kinase or atorvastatin. Blood Coagul Fibrinolysis. 2008;19:709–718. 18832915 10.1097/MBC.0b013e32830b2891 PMC2713681

[16] De Silva TM, Kinzenbaw DA, Modrick ML, et al Heterogeneous impact of ROCK2 on carotid and cerebrovascular function. Hypertension. 2016;68:809–817. 27432870 10.1161/HYPERTENSIONAHA.116.07430 PMC4982851

[17] Zanin-Zhorov A, Weiss JM, Nyuydzefe MS, et al Selective oral ROCK2 inhibitor down-regulates IL-21 and IL-17 secretion in human T cells via STAT3-dependent mechanism. Proc Natl Acad Sci U S A. 2014;111:16814–16819. 25385601 10.1073/pnas.1414189111 PMC4250132

[18] Zanin-Zhorov A, Weiss JM, Trzeciak A, et al Cutting edge: selective oral ROCK2 inhibitor reduces clinical scores in patients with psoriasis vulgaris and Normalizes skin pathology via concurrent regulation of IL-17 and IL-10. J Immunol. 2017;198:3809–3814. 28389592 10.4049/jimmunol.1602142 PMC5421306

[19] Diep DTV, Hong K, Khun T, et al Anti-adipogenic effects of KD025 (SLx-2119), a ROCK2-specific inhibitor, in 3T3-L1 cells. Sci Rep. 2018;8:2477. 29410516 10.1038/s41598-018-20821-3 PMC5802830

[20] Sasaki Y, Suzuki M, Hidaka H The novel and specific Rho-kinase inhibitor (S)-(+)-2-methyl-1-[(4-methyl-5-isoquinoline)sulfonyl]-homopiperazine as a probing molecule for Rho-kinase-involved pathway. Pharmacol Ther. 2002;93:225–232. 12191614 10.1016/s0163-7258(02)00191-2

[21] Rodriguez A-M, Elabd C, Delteil F, et al Adipocyte differentiation of multipotent cells established from human adipose tissue. Biochem Biophys Res Commun. 2004;315:255–263. 14766202 10.1016/j.bbrc.2004.01.053

[22] Wang T, Kang W, Du L, et al Rho-kinase inhibitor Y-27632 facilitates the proliferation, migration and pluripotency of human periodontal ligament stem cells. J Cell Mol Med. 2017;21:3100–3112. 28661039 10.1111/jcmm.13222 PMC5661246

[23] Liao JK, Seto M, Noma K Rho kinase (ROCK) inhibitors. J Cardiovasc Pharmacol. 2007;50:17–24. 17666911 10.1097/FJC.0b013e318070d1bd PMC2692906

[24] Lee S-H, Huang H, Choi K, et al ROCK1 isoform-specific deletion reveals a role for diet-induced insulin resistance. Am J Physiol Endocrinol Metab. 2014;306:E332–E343. 24326423 10.1152/ajpendo.00619.2013 PMC3920011

[25] Chun KH, Araki K, Jee Y, et al Regulation of glucose transport by ROCK1 differs from that of ROCK2 and is controlled by actin polymerization. Endocrinology. 2012;153:1649–1662. 22355071 10.1210/en.2011-1036 PMC3320261

[26] Chun KH, Choi KD, Lee DH, et al In vivo activation of ROCK1 by insulin is impaired in skeletal muscle of humans with type 2 diabetes. Am J Physiol Endocrinol Metab. 2011;300:E536–E542. 21189360 10.1152/ajpendo.00538.2010 PMC3064006

[27] Soliman H, Varela JN, Nyamandi V, et al Attenuation of obesity-induced insulin resistance in mice with heterozygous deletion of ROCK2. Int J Obes (Lond). 2016;40:1435–1443. 27163743 10.1038/ijo.2016.89

[28] Santos R, Ursu O, Gaulton A, et al A comprehensive map of molecular drug targets. Nat Rev Drug Discov. 2016;16:19–34. 27910877 10.1038/nrd.2016.230 PMC6314433

[29] Otto TC, Lane MD Adipose development: from stem cell to adipocyte. Crit Rev Biochem Mol Biol. 2005;40:229–242. 16126487 10.1080/10409230591008189

[30] Bunnell BA, Flaat M, Gagliardi C, et al Adipose-derived stem cells: isolation, expansion and differentiation. Methods. 2008;45:115–120. 18593609 10.1016/j.ymeth.2008.03.006 PMC3668445

[31] Zuk PA, Zhu M, Ashjian P, et al Human adipose tissue is a source of multipotent stem cells. Mol Biol Cell. 2002;13:4279–4295. 12475952 10.1091/mbc.E02-02-0105 PMC138633

[32] Moseti D, Regassa A, Kim W-K Molecular regulation of adipogenesis and potential anti-adipogenic bioactive molecules. Int J Mol Sci. 2016;17:124. 10.3390/ijms17010124 PMC4730365 26797605

[33] Summers SA, Whiteman EL, Birnbaum MJ Insulin signaling in the adipocyte. Int J Obesity. 2000;24:SS67–S70. 10.1038/sj.ijo.0801509 11126246

[34] Zhang HH, Huang J, Düvel K, et al Insulin stimulates adipogenesis through the Akt-TSC2-mTORC1 pathway. PLOS ONE. 2009;4:e6189. 19593385 10.1371/journal.pone.0006189 PMC2703782

